# Fluorescence detection of dopamine signaling to the primate striatum in relation to stimulus–reward associations

**DOI:** 10.1073/pnas.2426861122

**Published:** 2025-03-13

**Authors:** Gaoge Yan, Hidetoshi Amita, Satoshi Nonomura, Ken-ichi Inoue, Wolfram Schultz, Masahiko Takada

**Affiliations:** ^a^Systems Neuroscience Section, Center for the Evolutionary Origins of Human Behavior, Kyoto University, Inuyama, Aichi 484-8506, Japan; ^b^Department of Systems Physiology, Shiga University of Medical Science, Otsu, Shiga 520-2192, Japan; ^c^Department of Physiology, Development and Neuroscience, University of Cambridge, Cambridge CB2 3DY, United Kingdom

**Keywords:** caudate nucleus, putamen, reward prediction, fiber photometry, monkey

## Abstract

Dopamine (DA) signals to the striatum play critical roles in shaping and sustaining stimulus-reward associations. In primates, however, the dynamics of the DA signals remain unknown since conventional methods are not necessarily appropriate in terms of the spatiotemporal resolution or chemical specificity sufficient for detecting the DA signals. In our study, fiber photometry with a fluorescent DA sensor was employed to identify reward-related DA transients in the monkey striatum. This technique, which directly monitors local DA release, reveals a reward prediction error signal in the anterior putamen originating from midbrain DA neurons. Further, DA transients in the head of the caudate nucleus exhibit a value-based response to reward-predicting stimuli. These signals have been found to arise from two separate groups of DA neurons in the substantia nigra pars compacta. The present results demonstrate that fluorescence DA monitoring is applicable to detect DA signals in the primate striatum for investigating their roles.

## Significance

Reward-anticipatory behavior following a reward-predicting stimulus is achieved through Pavlovian conditioning. Dopamine (DA) released within the striatum, the main input station of the basal ganglia, plays a key role in this behavior. However, it remains unclear what type of DA signal is conveyed to the striatum in relation to stimulus-reward associations. To detect DA transients in the anterior striatal sectors responsible for the stimulus-reward associations, we applied fiber photometry with a fluorescent DA sensor to monkeys being engaged in a Pavlovian conditioning task. Our study demonstrates that this technique is useful to capture the DA transients in brain structures of the task-performing monkeys, and that the DA transients vary depending on the striatal territories.

Dopamine (DA), released within the striatum, plays a pivotal role in forming and sustaining stimulus-reward associations via the input derived from the substantia nigra (1–7). To detect the intrastriatal DA release, in vivo microdialysis and in vivo voltammetry have been applied in primates (8–16). However, these conventional methods are lacking in spatiotemporal resolution or chemical specificity sufficient for detecting rapid DA signals, respectively. Due to these technical limitations, only a limited number of studies have demonstrated the measurement of DA signals in the striatum of task-performing monkeys (12–16). Recently, newly developed fluorescent DA biosensors have gained an improved capacity to analyze the dynamics of DA signals with high temporal resolution (17, 18). These sensors engineered based on inert human DA receptors allow us to measure DA concentrations more precisely by changes in fluorescence intensity. This technique has attracted much attention to identify DA released within the striatum of rodents (19–22), but it has not as yet been applied to the primate brain.

In primates, the striatum is separated by the internal capsule into two distinct structures, known as the caudate nucleus and the putamen. Of particular interest is that the anterior parts of the striatum, i.e., caudate head and anterior putamen, are involved in stimulus-reward associations (23–27). According to previous studies, these striatal territories have massive projections from DA neurons in the medial aspect of the substantia nigra pars compacta (SNc) (28, 29), and the DA neurons therein exhibit a positive response to unpredicted reward delivery but a negative response to unpredicted reward omission, which aligns with the concept of reward prediction error (RPE) in reinforcement learning (30–34). Taken together, we suppose that DA signals transmitted to the anterior striatum correspond to the RPE.

In the present study, an attempt was made to capture DA transients in the primate striatum in relation to stimulus-reward associations. We employed fiber photometry with a fluorescent DA sensor, termed dLight, for detecting DA release, and successfully monitored DA transients in the anterior striatum during Pavlovian conditioning. Our data show that DA transients in the anterior putamen signal the RPE in response to outcomes, whereas those in the caudate head exhibit a value-based response to reward-predicting stimuli.

## Results

### Anticipatory Behavior during Pavlovian Conditioning.

**Fig. 1.**
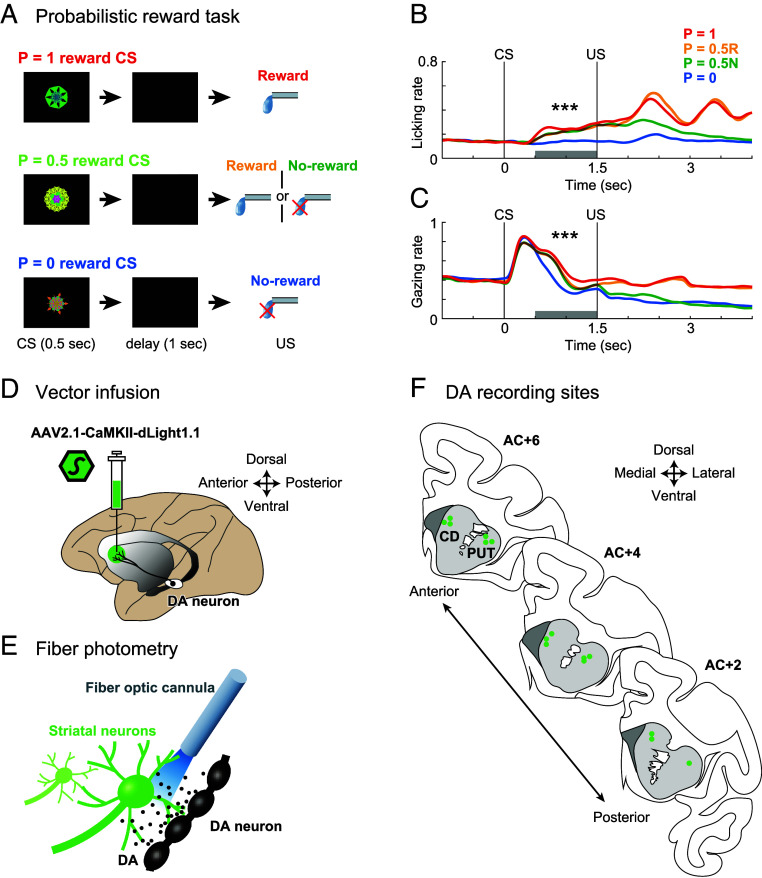
DA recording in the striatum using a fluorescent sensor during Pavlovian conditioning. (*A*) Sequence of events in the probabilistic reward task. Three different CSs were associated with three different reward probabilities, respectively: P = 1, P = 0.5, and P = 0. (*B*) Licking rates in the probabilistic reward task. Red and blue lines indicate average licking rates for CSs for P = 1 reward and P = 0 reward, respectively. Yellow and green lines indicate trials with CS predicting reward with P = 0.5 that was followed by reward and no-reward, respectively. The gray bar represents 1-s statistical analysis window (N = 2 subjects, n = 74 sessions; Dunnett’s test; P = 0 vs. 1: *P* = 2.3 × 10^−12^; P = 0 vs. 0.5: *P* = 1.9 × 10^−7^). ***P < 0.001. (*C*) Gazing rates in the probabilistic reward task. The gray bar represents 1-s statistical analysis window (N = 2 subjects, n = 56 sessions; Dunnett’s test; P = 0 vs. 1: *P* = 8.6 × 10^−4^; P = 0 vs. 0.5: *P* = 0.013). Other conventions are as in *B*. (*D*) Schema showing vector infusion into the striatum for expressing a fluorescent DA biosensor (dLight1.1). See also Table 1 and *SI Appendix*, Fig. S1. (*E*) Fiber photometry to capture DA transients in the striatum using a fiber optic cannula. (*F*) Reconstruction of DA recording sites on coronal sections 6, 4, and 2 mm anterior to the anterior commissure (AC+6, AC+4, and AC+2, respectively). Green circles denote the recording sites in three overlaid sections taken from the two animals (monkeys CR and DO). CD, caudate head; PUT, anterior putamen.

In this study, we used a Pavlovian conditioning task with probabilistic reward. The probabilistic reward task served to investigate DA signals during established task performance (Fig. 1*A*). Conditioned stimuli (CSs) were paired with three different reward probabilities (i.e., P = 1 reward CS, P = 0.5 reward CS, and P = 0 reward CS). A liquid reward was used as an unconditioned stimulus (US). Each CS was presented at the center of a display monitor for 0.5 s. Following a delay of 1 s, either reward or no-reward was delivered. After training for 2 mo, the licking rate (number of trials with licking the spout/all trials) of the monkeys after the CS reflected the expected value (i.e., sum of reward amount × reward probability) (Fig. 1*B*, Dunnett’s test; P = 0 vs. 1: *P* = 2.3 × 10^−12^, *d* = 1.3, 95% CI [0.95, 1.6]; P = 0 vs. 0.5: *P* = 1.9 × 10^−7^, *d* = 0.95, 95% CI [0.71, 1.24]). A similar result was seen with gazing rate (number of trials with gazing at the center/all trials) (Fig. 1*C*, Dunnett’s test; P = 0 vs. 1: *P* = 8.6 × 10^−4^, *d* = 0.70, 95% CI [0.53, 0.90]; P = 0 vs. 0.5: *P* = 0.013, *d* = 0.53, 95% CI [0.38, 0.70]). Both behavioral results were consistent with those of previous studies (35–39).

### DA Recording in the Striatum Using a Fluorescent DA Sensor.

First, we verified the expression of a fluorescent DA sensor (dLight1.1) in the rat striatum through local infusion of an adeno-associated virus (AAV) vector (*SI Appendix*, Fig. S1*A*) and detected the fluorescence signal in the ventral striatum during Pavlovian conditioning (*SI Appendix*, Fig. S1*B*). Second, to express the dLight1.1 in the monkey striatum, we delivered the same vector via local infusion (Fig. 1*D* and Table 1). Six weeks following the vector infusion, we started photometry recordings using a fiber optic cannula (Fig. 1*E*). After completion of the recordings, we histologically confirmed the expression of dLight1.1 in the striatum (*SI Appendix*, Fig. S1*C*). The recording sites were located primarily in the anterior parts of the dorsal striatum (i.e., the caudate head and the anterior putamen) (Fig. 1*F*).

**Table 1.** Subject and vector information

Subject	Species	Sex	Age	Vector	Titer (gc/mL)	Volume (µL/site)	Injection tracks
CR	*Macaca mulatta*	M	6	AAV2.1-CaMKIIa-dLight1.1	2.0 × 10^13^	2.5	2 tracks in CD (Lt) 2 tracks in PUT (Lt)
DO	*Macaca mulatta*	M	7	AAV2-CaMKIIa-dLight1.1	2.0 × 10^13^	2.5	3 tracks in CD (Rt) 3 tracks in PUT (Rt)
				AAV2.1-CaMKIIa-dLight1.1	2.0 × 10^13^	2.5	3 tracks in CD (Lt) 3 tracks in PUT (Lt)
CN	*Macaca fuscata*	M	12	rAAV2-retro-hSyn-mScarlet	4.0 × 10^13^	2.5	1 track in CD (Lt)
				rAAV2-retro-hSyn-AcGFP	4.0 × 10^13^	2.5	1 track in PUT (Lt)
DK	*Macaca mulatta*	F	18	rAAV2-retro-hSyn-mScarlet	4.0 × 10^13^	2.5	1 track in PUT (Lt)
				rAAV2-retro-hSyn-AcGFP	4.0 × 10^13^	2.5	1 track in CD (Lt)

Fluorescence DA recording was performed in two macaques (monkeys CR and DO), and retrograde neuron labeling was carried out in other two macaques (monkeys CN and DK). M, Male; F, Female; CD, caudate head; PUT, anterior putamen; Lt, Left hemisphere; Rt, Right hemisphere.

### DA Signaling to the Monkey Striatum in the Probabilistic Reward Task.

**Fig. 2.**
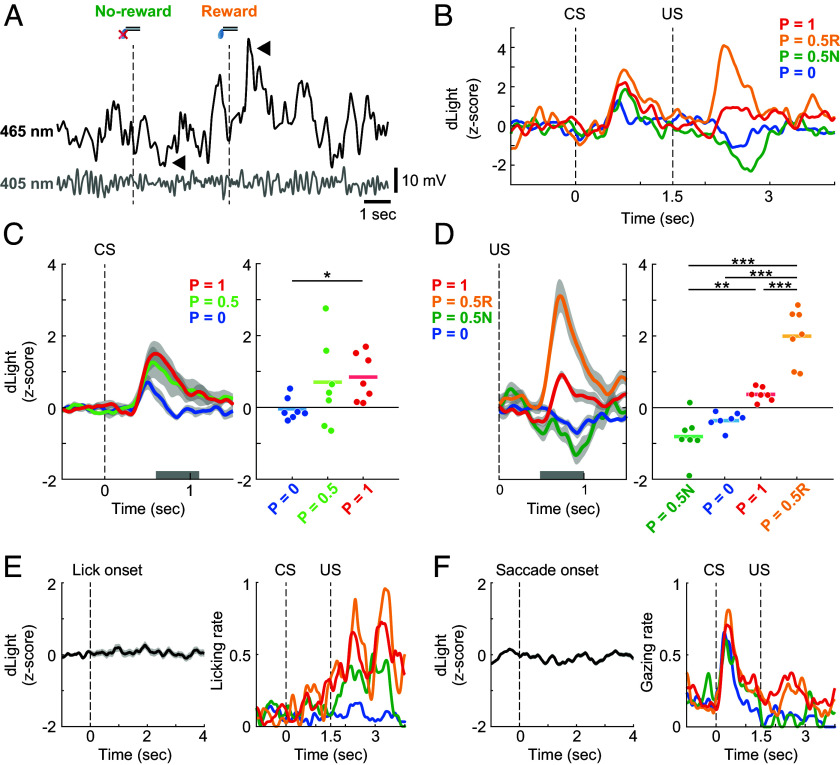
dLight fluorescence in the anterior putamen. (*A*) Example fluorescence traces in the anterior putamen (PUT) during the probabilistic reward task. Each dashed line indicates the timings of US presentations following P = 0.5 reward CS. Black and gray traces denote 465-nm dLight signal and 405-nm isosbestic signal, respectively. Arrowheads point to fluorescence changes in response to the US presentations (no-reward and reward). (*B*) Representative dLight signal in PUT in a single recording session. Red, yellow, green, and blue lines denote reward trials following P = 1 reward CS P = 1), reward trials following P = 0.5 reward CS (P = 0.5R), no-reward trials following P = 0.5 reward CS (P = 0.5N), and no-reward trials following P = 0 reward CS (P = 0), respectively. Dashed lines indicate the timings of CS and US presentations. (*C*, *Left*) Average normalized dLight signal in PUT in response to three CS types (P = 1, 0.5, and 0). Red, light green, and blue lines denote P = 1 reward CS, P = 0.5 reward CS, and P = 0 reward CS trials, respectively. Shaded areas denote mean ± SEM. The gray bar represents a 0.5-s statistical analysis window. (*Right*) Responses to three CS types in a 0.5-s window (n = 7 sessions; Dunnett’s test; P = 0 vs. 1: *P* = 0.047; P = 0 vs. 0.5: *P* = 0.10). Each bar indicates mean. *P < 0.05. (*D*, *Left*) Average normalized dLight signal in PUT in response to US among four trial conditions (P = 1, 0.5R, 0.5N, and 0). Shaded areas denote mean ± SEM. The gray bar represents a 0.5-s statistical analysis window. (*Right*) Responses to US in the 0.5-s window (n = 7 sessions; Tukey’s test; P = 0.5R vs. 1: *P* = 2.0 × 10^−5^; P = 0.5R vs. 0: *P* = 6.4 × 10^−8^; P = 0.5R vs. 0.5N: *P* = 2.3 × 10^−8^; P = 1 vs. 0: *P* = 0.059; P = 1 vs. 0.5N: *P* = 0.0012; P = 0 vs. 0.5N: *P* = 0.38). Each bar indicates mean. **P < 0.01, ***P < 0.001. (*E*, *Left*) Representative dLight signal in PUT aligned by the lick onset (n = 378 times) in a single recording session. The shaded area denotes mean ± SEM. The dashed line indicates the initiation of licking movement. (*Right*) Licking rates in a recording session. All conventions are as in Fig. 1*B*. (*F*, *Left*) Representative dLight signal in PUT aligned by the saccade onset (n = 987 times) in a single recording session. The shaded area denotes mean ± SEM. The dashed line indicates the initiation of saccadic eye movement. (*Right*) Gazing rates in a recording session. All conventions are as in Fig. 1*C*.

We recorded fluorescence signals in the anterior striatum while the monkeys were performing the probabilistic reward task. The 465-nm dLight signal in the anterior putamen was changed by US presentation, while the 405-nm isosbestic signal was not changed (Fig. 2*A*). In the probabilistic reward task, a typical dLight fluorescence signal in the anterior putamen exhibited transient responses to both CS and US (Fig. 2*B*). Here, we compared population responses to three CSs, namely CSs for rewards delivered with P = 1 (always reward), P = 0.5, and P = 0 (never reward) during the delay period after the presentation of CSs (0.1 to 0.6 s from CS offset) (Fig. 2*C*). The population signals in response to the P = 1 reward CS were significantly greater than those in response to the P = 0 reward CS (Dunnett’s test; *P* = 0.047, *d* = 1.7, 95% CI [0.61, 3.7]). During the post-US delivery period (0.5 to 1.0 s after US onset; US period), we compared population responses to US among four trial conditions, namely reward following the P = 1 reward CS (P = 1), reward following the P = 0.5 reward CS (P = 0.5R), no-reward following the P = 0.5 reward CS (P = 0.5N), and no-reward following the P = 0 reward CS (P = 0) (Fig. 2*D*). The population signals in response to reward following the P = 0.5 reward CS were significantly greater than those in the other three conditions (Tukey’s test; P = 0.5R vs. 1: *P* = 2.0 × 10^−5^, *d* = 2.5, 95% CI [1.1, 5.2]; P = 0.5R vs. 0: *P* = 6.4 × 10^−8^, *d* = 3.6, 95% CI [1.8, 7.3]; P = 0.5R vs. 0.5N: *P* = 2.3 × 10^−8^, *d* = 3.5, 95% CI [1.6, 7.2]). By contrast, the response to reward omission following the P = 0.5 reward CS was significantly below the baseline (one-sample *t* test; *P* = 0.012, mean difference = −0.80, 95% CI [−1.3, −0.24]). The population responses to the reward following the P = 1 reward CS and no-reward following the P = 0 reward CS were in between. Though there was no significant difference between these two conditions (Tukey’s test; *P* = 0.059, *d* = 3.4, 95% CI [1.5, 7.0]), the response to the reward following the P = 1 reward CS was above the baseline (one-sample *t* test; *P* = 0.002, mean difference = 0.36, 95% CI [0.19, 0.54]) and the response to the no-reward following the P = 0 reward CS was below the baseline (one-sample *t* test; *P* = 0.002, mean difference = −0.36, 95% CI [−0.53, −0.19]). These weak but evident responses to the predicted outcomes may at least partly be due to the variation in reward delivery timing among trials. We confirmed that the dLight signals in the anterior putamen were not related to either eye movement or licking movement (Fig. 2 *E* and *F*). These findings indicate that DA transients in the anterior putamen reported both positive and negative RPEs to US, which are generally consistent with the bidirectional electrophysiological impulse responses of DA neurons to RPEs (35–38). These data demonstrate successful recordings of fluorescence DA signals in monkey striatum using fiber photometry.

**Fig. 3.**
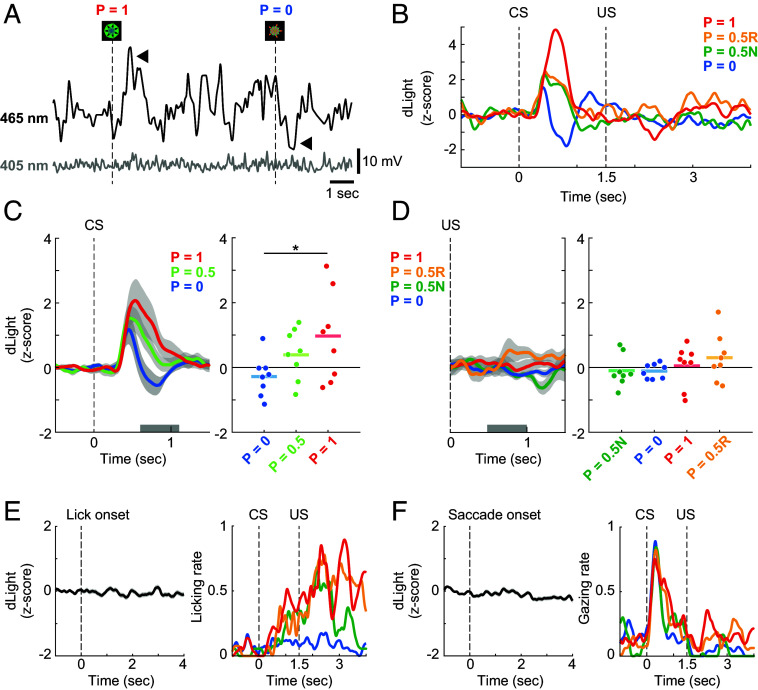
dLight fluorescence in the caudate head. (*A*) Example fluorescence traces in the caudate head (CD) during the probabilistic reward task. Each dashed line indicates the timings of CS presentations. Black and gray traces denote 465-nm dLight signal and 405-nm isosbestic signal, respectively. Arrowheads point to fluorescence changes in response to CS presentations (P = 1 reward CS and P = 0 reward CS). (*B*) Representative dLight signal in CD in a single recording session. All conventions are as in Fig. 2*B*. (*C*, *Left*) Average normalized dLight signal in CD in response to three CS types (P = 1, 0.5, and 0). (*Right*) Responses to three CS types in a 0.5-s window (n = 8 sessions; Dunnett’s test; P = 0 vs. 1: *P* = 0.014; P = 0 vs. 0.5: *P* = 0.22). All conventions are as in Fig. 2*C*. (*D*, *Left*) Average normalized dLight signal in CD in response to US among four trial conditions (P = 1, 0.5R, 0.5N, and 0). (*Right*) Responses to US in a 0.5-s window (n = 8 sessions; Tukey’s test; P = 0.5R vs. 1: *P* = 0.80; P = 0.5R vs. 0: *P* = 0.46; P = 0.5R vs. 0.5N: *P* = 0.49; P = 1 vs. 0: *P* = 0.93; P = 1 vs. 0.5N: *P* = 0.95; P = 0 vs. 0.5N: *P* = 1.0). All conventions are as in Fig. 2*D*. (*E*, *Left*) Representative dLight signal in CD aligned by the lick onset (n = 437 times) in a single recording session. (*Right*) Licking rates in a recording session. All conventions are as in Fig. 2*E*. (*F*, *Left*) Representative dLight signal in CD aligned by the saccade onset (n = 849 times) in a single recording session. (*Right*) Gazing rates in a recording session. All conventions are as in Fig. 2*F*.

On the other hand, the 465-nm dLight signal in the caudate head was clearly changed by CS presentation, while the 405-nm isosbestic signal was not changed (Fig. 3*A*). A dLight fluorescence signal in the caudate head typically showed transient responses to the CSs, but weak responses to the US (Fig. 3*B*). During the delay period after the CS presentation, the population signals following the P = 1 reward CS were significantly greater than those after the P = 0 reward CS (Fig. 3*C*, Dunnett’s test; *P* = 0.014, *d* = 1.2, 95% CI [0.24, 2.7]). Following the US, however, the signals displayed no significant differences among the four trial conditions (Fig. 3*D*, Tukey’s test; P = 0.5R vs. 1: *P* = 0.80, *d* = 0.32, 95% CI [−0.72, 1.5]; P = 0.5R vs. 0: *P* = 0.46, *d* = 0.67, 95% CI [−0.27, 1.9]; P = 0.5R vs. 0.5N: *P* = 0.49, *d* = 0.57, 95% CI [−0.66, 2.1]; P = 1 vs. 0: *P* = 0.93, *d* = 0.30, 95% CI [−0.76, 1.5]; P = 1 vs. 0.5N: *P* = 0.95, *d* = 0.23, 95% CI [−1.1, 1.7]; P = 0 vs. 0.5N: *P* = 1.0, *d* = 0.027, 95% CI [−1.1, 1.2]), unlike the fluorescence signals in the anterior putamen (Fig. 2*D*). We confirmed that the dLight signals in the caudate head were not related to either eye movement or licking movement (Fig. 3 *E* and *F*). These results indicate that DA transients in this region were evoked by the visual, reward-predicting CS but not by outcome itself (US).

**Fig. 4.**
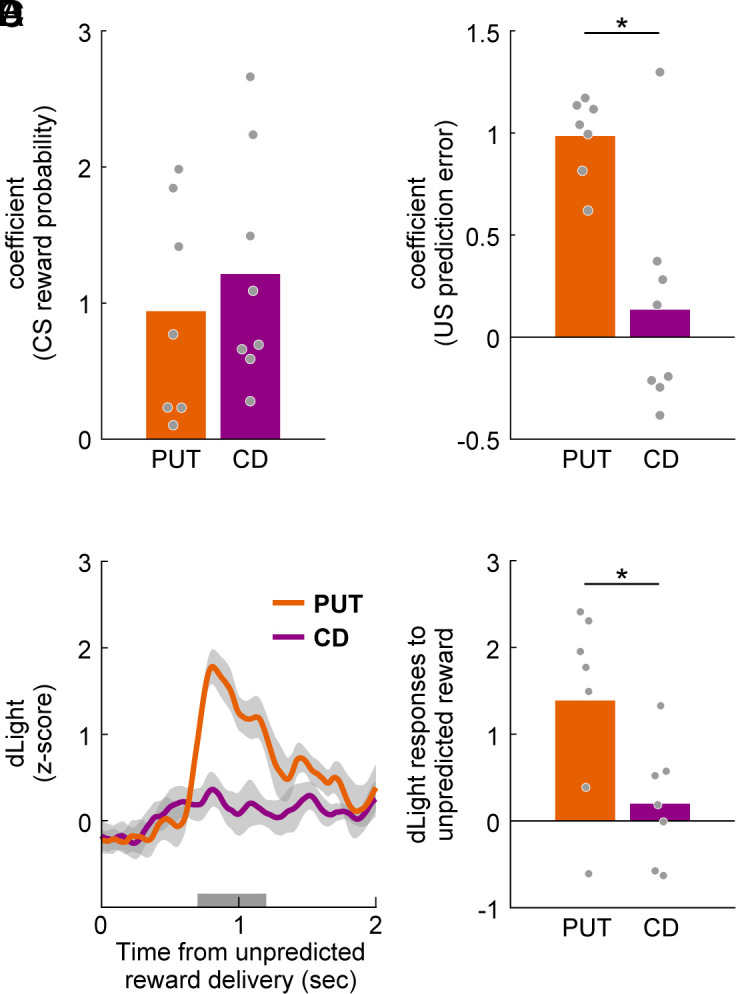
Comparison of dLight fluorescence between the anterior putamen and the caudate head. (*A*) Regression coefficients for CS reward probability in the probabilistic reward task. Orange and purple bars indicate average coefficients of dLight signals in PUT and CD, respectively (Wilcoxon rank-sum test; *P* = 0.46). Each gray circle denotes each recording session. (*B*) Regression coefficients for US prediction error in the probabilistic reward task. Orange and purple bars indicate average coefficients of dLight signals in PUT and CD, respectively (Wilcoxon rank-sum test; *P* = 0.014). *P < 0.05. (*C*) Average normalized dLight signal in response to unpredicted reward delivery. Orange and purple lines indicate the dLight signals in PUT and CD, respectively (PUT: n = 7 sessions, CD: n = 7 sessions). Shaded areas denote mean ± SEM. The gray bar represents a 0.5-s statistical analysis window. (*D*) dLight responses to unpredicted reward in the 0.5-s window (two sample *t* test; *P* = 0.032). Orange and purple bars indicate the dLight responses in PUT and CD, respectively. *P < 0.05.

Next, to compare the dLight responses to CS between the anterior putamen and the caudate head, we performed linear regression analysis of CS responses with reward probability for each session. The regression coefficients were positive in both the anterior putamen and the caudate head, and displayed no significant difference between these two regions (Fig. 4*A*, Wilcoxon rank-sum test; *P* = 0.45, *r* = 0.19, 95% CI [−1.2, 0.66]). By contrast, linear regression analysis of US responses with prediction error for each session displayed a significantly higher regression coefficient in the anterior putamen than in the caudate head (Fig. 4*B*, Wilcoxon rank-sum test; *P* = 0.014, *r* = 0.61, 95% CI [0.68, 3.1]). This suggests that the DA transients in response to US with prediction error are different in these two regions.

### DA Signaling Related to Unpredicted Reward.

To investigate whether DA transients show a positive response to unpredicted reward, we recorded a dLight fluorescence signal during unpredicted reward delivery. The population fluorescence signals in the anterior putamen exhibited a positive response to the unpredicted reward, while those in the caudate head had a weak response to the unpredicted reward (Fig. 4*C*). The dLight responses to the unpredicted reward displayed a significant difference between the anterior putamen and the caudate head (Fig. 4*D*, two-sample *t* test; *P* = 0.032, *d* = 1.2, 95% CI [0.10, 2.3]). These results (Fig. 4 *B* and *C*) suggest that DA transients in the anterior putamen may encode a clearer prediction error to US than in the caudate head.

### Projections of Distinct Populations of DA Neurons to the Caudate Head vs. the Anterior Putamen.

**Fig. 5.**
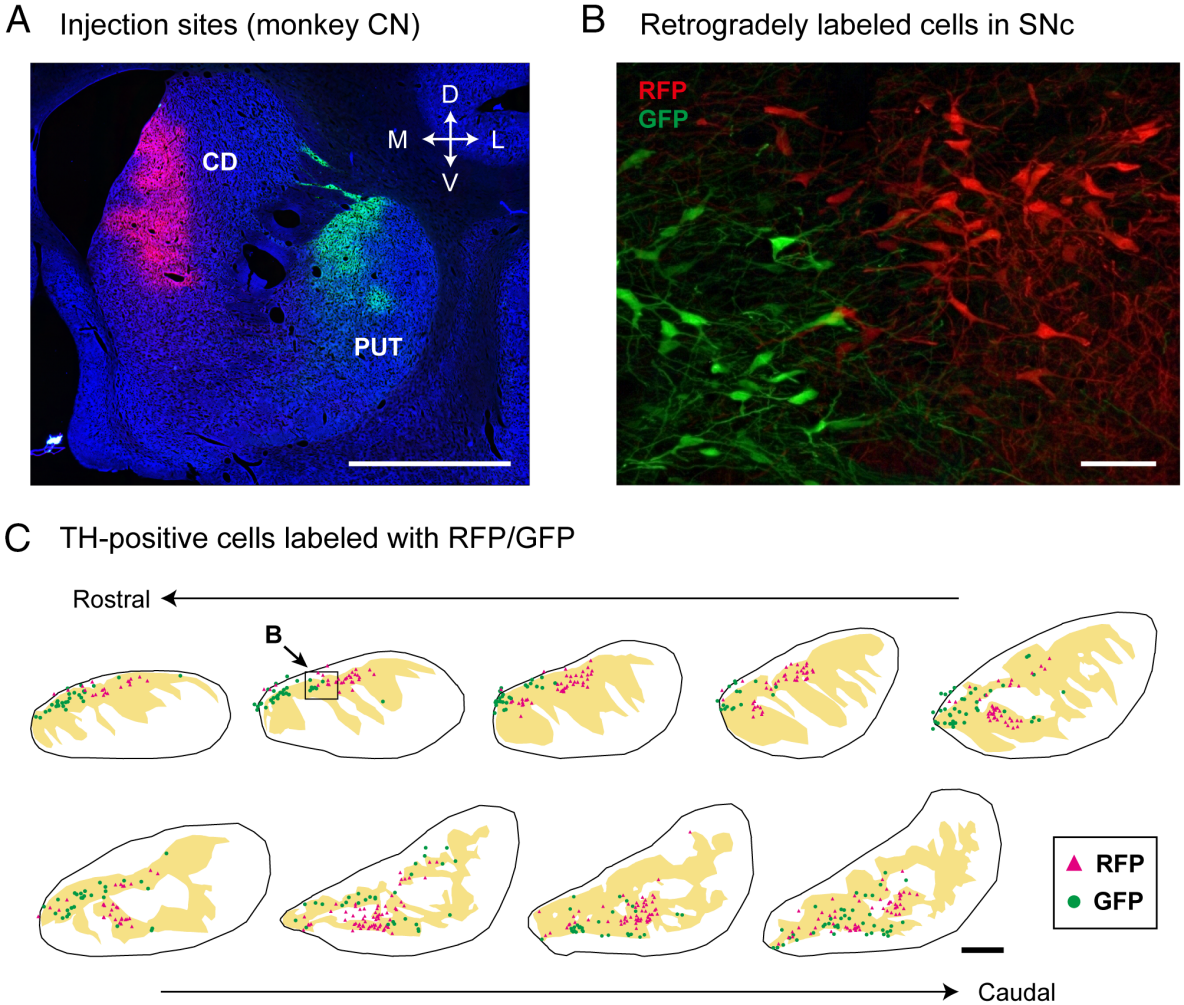
Projections of distinct populations of DA neurons to the caudate head vs. the anterior putamen. (*A*) Sites of vector injections in a representative coronal section (monkey CN). rAAV2-retro-RFP (red) and rAAV2-retro-GFP (green) vectors were injected into CD and PUT, respectively. (Scale bar, 5 mm.) (*B*) CD-projecting and PUT-projecting cells in the SNc that were labeled with RFP (red) and GFP (green), respectively. (Scale bar, 100 µm.) (*C*) Distribution of TH-positive cells in SNc labeled with RFP (red) or GFP (green). The yellow shaded area demarcates the SNc. Coronal sections 500-μm apart are arranged rostrocaudally. The rectangle pointed to by *B* corresponds to the area shown in *B*. (Scale bar, 1 mm.) See also *SI Appendix*, Fig. S2.

Our fluorescence DA recordings indicate that DA signals conveyed to caudate head and anterior putamen are different from each other. To validate whether caudate head and anterior putamen receive projections from distinct populations of DA neurons, we injected dual-color retrograde AAV vectors (rAAV2-retro) into individual striatal territories. In monkey CN, one vector expressing red fluorescent protein (RFP) was injected into the caudate head, while the other expressing green fluorescent protein (GFP) was injected into the anterior putamen (Fig. 5*A*). The injection sites largely corresponded to the regions in which the fluorescence DA signals were recorded (Fig. 1*F*). Cells retrogradely labeled with RFP from the caudate head and GFP from the anterior putamen (Fig. 5*B*) were both localized in the medial part of SNc (Fig. 5*C*), and they were also immunostained for tyrosine hydroxylase (TH). We then confirmed that the RFP-labeled cells were spatially segregated from the GFP-labeled cells, and that virtually none of them were double-labeled with the two tracers. In another case (monkey DK), the site of vector injection in the caudate head was situated more ventrally than in monkey CN (*SI Appendix*, Fig. S2*A*), and the injection site in the anterior putamen was similar to that in monkey CN. Consistent with the findings in monkey CN, the two populations of retrogradely labeled cells were both located in the medial part of SNc, although their distributions were spatially separated from each other (*SI Appendix*, Fig. S2*B*).

## Discussion

We successfully monitored DA transients in the monkey striatum using fiber photometry with the fluorescent DA biosensor, dLight1.1. In the present study, motion artifacts were negligible in our head-fixed condition, as shown by the 405-nm isosbestic signal (Figs. 2*A* and 3*A*). By contrast, the 465-nm dLight signal dynamically changed based on events including CS presentation and reward delivery (Figs. 2*A* and 3*A*), though it was difficult to confirm sufficient DA transients in real time due to fluctuations in the baseline signals. The changes observed in dLight signals in the anterior putamen and caudate head were not related to either eye movement or licking movement (Figs. 2 *E* and *F* and 3 *E* and *F*). The reason that the changes related to stimulus-reward associations could be ascribed to our recording sites that were located in the striatal association territory (40). Similar to the activity of midbrain DA neurons (41, 42), the dLight signal in the anterior striatum showed a positive response to an unpredicted reward acquisition and a negative response to an unpredicted reward omission (Fig. 2*D*). This indicates that the present methodology permits the detection of both increment and decrement in DA release, while it is more challenging to detect reduced DA release using in vivo voltammetry. Previous studies using voltammetry suggest that DA transients in the monkey striatum respond to CS associated with probabilistic rewards and unpredicted outcomes (12–14), which has difficulty in distinguishing DA from norepinephrine (43, 44). These results were reconfirmed by our study using dLight, which has a higher molecular specificity for DA than other substances (17). Moreover, our fiber photometry has the ability to easily access deep brain structures, such as the monkey striatum, because optical probes used for fiber photometry are more durable than carbon probes for voltammetry. It should be noted here, however, that the response latencies to CS in the present study were 50 to 100 ms slower than those in previous rodent studies (19, 21, 22, 45, 46). Because the delayed latency was seen in the rat striatum (*SI Appendix*, Fig. S1), this would not be due to the species difference between rodents and monkeys. Such distinct response latencies may be accounted for by the variation of DA biosensors (e.g., dLight1.1, dLight1.2, dLight1.3b, and GRAB-DA2m). Another reason may lie in the possible difference in data analysis. In the previous works, the dLight signals were often downsampled and smoothed with a moving average or a median filter to improve the signal-to-noise ratio (19, 22, 47). Therefore, the dLight response latency in the previous studies might not completely be accurate. By contrast, we did not apply these smoothing methods to our data. Further investigations are required to determine whether this delayed latency is due to the kinetics of DA biosensors or other reasons (48, 49).

Surprisingly, our data have demonstrated the distinct patterns of DA signaling to the anterior putamen and the caudate head (Fig. 4). It seems unlikely that the differential behavioral or learning performance may have caused the difference in DA transients between these two regions, because no clear differences were observed in licking or eye movement in response to CS or US between these two recording sessions (Figs. 2 *E* and *F* and 3 *E* and *F*). Earlier experiments using voltammetry have also shown that DA transients vary depending on the striatal territories recorded (12, 13, 15). However, this seems contradictory to the activity of typical midbrain DA neurons. Previous electrophysiological studies have reported that the DA neuron activity corresponds to a temporal-difference error (TD error) in reinforcement learning (31). If the DA signal in the anterior striatum follows this TD error model (30, 50), the response magnitudes to CS should be consistent with the reward probabilities of CS (P = 1 > 0.5 > 0), and those to US should be consistent with the RPE in US epoch (P = 0.5 with reward > 1 = 0 > 0.5 followed by reward omission). However, the present data showed that the DA signal in the caudate head exhibited a clear CS response, that was largely consistent with the TD error, but not a clear US response. This DA signal in the caudate head is partially aligned with the activity of DA neurons exhibiting no clear response to unpredicted reward outcomes, as reported in a previous study (51) in which these DA neurons project to the caudate tail, but not to the caudate head. In prior electrophysiological works, reward-insensitive DA neurons may have been overlooked, because reward responses are often used as a criterion for identifying DA neurons. Therefore, future study is needed to clarify whether DA neurons, which do not exhibit a reward response, project to the caudate head. Conversely, the DA signal in the anterior putamen displayed a clear US response in register with the TD error, but had a weaker CS response. The observed heterogeneity between the caudate head and the anterior putamen implies that two components of the TD error-based RPE signal for CS and US epochs may be segregated within the striatum. This heterogeneity is at least partly similar to that of DA signals between the dorsomedial striatum (DMS) and the dorsolateral striatum (DLS), as reported in recent rodent studies (22, 46) in which DA responses to CS in the DMS are clearer than in the DLS. Other studies demonstrated that DA signaling to the most dorsal part of DMS exhibited a weak response to US (52, 53). There are three possible mechanisms contributing to such heterogeneity of DA signals in the striatum. First, differential responses to CS vs. US may reflect a certain heterogeneity of RPE responses, similar to the asymmetric scaling of positive vs. negative RPEs encoded by midbrain DA neurons (54). Second, the heterogeneity of DA responses detected in the present study may result from presynaptic modulation of nigrostriatal DA transmission mediated through presynaptic acetylcholine and/or glutamate receptors on DA terminals (55–57). Third, diverse subsets of midbrain DA neurons may transmit distinct signals to different striatal territories, similar to the dissociation between the DMS and the DLS in rodents (58, 59). It should also be emphasized that several studies support the heterogeneity of DA functions (60–62). Further investigations are called for to identify activity of DA neurons projecting to each striatal territory, explore DA transients taken from the entire striatum by widespread dLight expression, and record both DA neuron activity and corresponding dLight signal from the striatum simultaneously.

The present results confirm that DA neurons are segregated in their projections to distinct territories of the striatum, i.e., caudate head vs. anterior putamen (Fig. 5*C*), in agreement with previous studies (28, 29, 63–65). It has been reported that midbrain DA neurons projecting to different striatal territories are differentially distributed (40, 51, 63, 64), and that there are molecular and functional differences in several regions of the midbrain in humans and monkeys (37, 64, 66–70). This favors our findings that separate populations of DA neurons in the substantia nigra convey heterogeneous signals to the caudate head and the anterior putamen. Further studies on primates are needed to explore DA functions in specific striatal regions for understanding the pathophysiology of Parkinson’s disease.

In conclusion, the present study demonstrates that the fiber photometry using dLight1.1 is applicable to capture the DA transients in the primate striatum. Therefore, the developed fiber photometry technique is widely suitable for identifying the physiological functions of DA and other substances in deep brain structures of task-performing monkeys.

## Materials and Methods

### Subjects.

Three male and one female macaques (*Macaca mulatta* and *Macaca fuscata*, 6 to 18 y old, 6.7 to 11.8 kg) were used for the present study (Table 1). All animal care and experimental procedures were approved by the Institutional Animal Care and Use Committee of Primate Research Institute, Kyoto University (Permission Number: 2021-020, 2022-041, 2023-138), and complied with the Guidelines for Care and Use of Nonhuman Primates (Primate Research Institute, Kyoto University) and the Guidelines for the Care and Use of Nonhuman Primates in Neuroscience Research (Japan Neuroscience Society).

### Surgical Procedures.

Each subject was initially sedated with ketamine hydrochloride (5 mg/kg, i.m.) and xylazine hydrochloride (0.5 mg/kg, i.m.), and anesthetized using an inhalational anesthetic (Isoflurane; 1 to 3%; Mylan Inc.). Under general anesthesia and sterile surgical conditions, a plastic head holder was implanted for each subject. The head holder was embedded in dental acrylic resin (Unifast II, GC Corporation) and secured to the skull with plastic anchor screws (M3, 6-mm and 8-mm long). Before and after the surgery, analgesics (Cefdinir; 0.5 mg, p.o. and Buprenorphine hydrochloride; 0.2 mg, i.m.) and an antibiotic (Ceftazidime; 25 mg/kg, i.v.) were administered. During surgery, the subject’s hydration was maintained with lactated Ringer’s solution (i.v.). For the health and well-being of subjects, the head post was routinely cleaned by flushing with saline solution. Training began after a 6-wk recovery period. An antibiotic ointment (Chlomy-P) was applied as needed.

### Behavioral Procedures.

Behavioral control and data acquisition were conducted by MATLAB-based software tool, NIMH onkeyLogic (https://monkeylogic.nimh.nih.gov/) provided by the National Institute of Mental Health. Output control was performed using a multifunction I/O device (PCIe-6323; National Instruments). The subject sat in a primate chair with its head fixed facing a monitor display (P2722H; Dell) in a sound-attenuated and electrically shielded room (71). Eye position was sampled at 400 Hz using an eye tracker system (ViewPoint MCU-400; Arrington Research). Liquid reward was delivered through a spout device placed in front of the subject’s mouth using a smoothflow pump (QI-100-TT-P-S; Tacmina), and its spout-licking behavior was detected by an infrared sensor (E3X-HD11; Omron) attached to the spout device.

### Probabilistic Reward Task.

Two subjects (monkeys CR and DO) were conditioned in a probabilistic reward task with a Pavlovian procedure (Fig. 1*A*). A total of six fractal objects were presented to the subject as CS, each predicting the outcome in a probabilistic manner. Two CSs were paired with reward delivery with a probability of P = 1 (P = 1 reward CS). Two other CSs were paired with reward delivery with P = 0.5 (P = 0.5 reward CS). The remaining two CSs were paired with P = 0 (no-reward; P = 0 reward CS). Each CS was pseudorandomly chosen and presented at the center of the screen for 0.5 s. Following a delay period of 1 s, either a reward accompanied by a tone or no-reward accompanied by a beep sound was presented based on CS. All trials were followed by a variable intertrial interval ranging from 3 to 7 s.

After a training period of 2 mo, we recorded the behavior and fluorescence signal while the subject was engaged in the behavioral task.

### Behavioral Data Analysis.

Analysis of licking and gazing behaviors was performed using MATLAB (MathWorks). The licking rate was calculated by dividing the number of trials in which a subject licked the spout by the total number of trials. The gazing rate was calculated by dividing the number of trials in which the subject’s eye position was within a central window (8° circle) of the screen by the total number of trials.

For the probabilistic reward task (Fig. 1 *B* and *C*), we plotted the dynamics of licking rates and gazing rates across sessions for four trial types (P = 1 reward CS, P = 0.5 reward CS with reward, P = 0.5 reward CS without reward, and P = 0 reward CS). In the statistical analysis, we calculated the mean licking rate and mean gazing rate within a 1-s time window from CS offset to US onset (i.e., delay period) for each CS type (P = 1, P = 0.5 and P = 0 reward CSs, respectively) in each session. Dunnett’s test was used to compare the licking rates and gazing rates in response to P = 0 reward CS with those in response to P = 0.5 or P = 1 reward CS.

### Identification of Striatal Regions.

A plastic recording chamber was connected to the head holder with dental acrylic resin under general anesthesia and sterile surgical conditions. The recording chamber was placed for targeting the caudate head and the anterior putamen, then a craniotomy was performed based on a rhesus monkey brain atlas (72). We routinely cleaned the chamber by flushing with saline solution or a mixture of betadine and saline solution at least three times a week. After 2 wk of recovery period, in order to identify the locations of task-related striatal regions, we started the recording of neuronal activity from the caudate head and anterior putamen using an epoxy-coated tungsten microelectrode (150-μm thick; FHC Inc.). Each recording site was targeted by MR images using a 0.3T permanent MRI scanner (AIRIS Vento; Fujifilm) and a grid system with 1-mm spacing. The electrode was inserted and advanced through a stainless-steel guide tube by an oil-driven micromanipulator (MO-97; Narishige). The activities of striatal neurons were amplified using a neurodigitizer amplifier (PZ5-32; Tucker Davis Technologies) and sampled using a real-time signal processor (RZ5D; Tucker Davis Technologies) running the Synapse software suite (bandpass-filter: 0.6 to 8 kHz).

### Fiber Photometry.

After identifying the locations of task-related striatal regions by electrophysiology, a custom-made injectrode consisting of a fused silica tubing (outer/inner diameter: 150/75 μm; Polymicro Technologies) and a tungsten microelectrode (200-μm thick; FHC Inc.) was used for vector infusion (71). The distance between the tips of the electrode and the beveled silica tubing was 500 μm. Each injection site was determined based on the preceding electrophysiological recording data and infused the vector into the striatal regions (i.e., caudate head and anterior putamen) for each subject. As viral vector, we used AAV2.1-CaMKIIα-dLight1.1 (73) or AAV2-CaMKIIα-dLight1.1 (2.0 × 10^13^ genome copies/mL) (Table 1). The vector was infused at a speed of 0.1 μL/min (2.5 μL/site, two sites/track) using a 10-μL Hamilton syringe (Model 701 RN Syringe #7635-01; Hamilton) with a manual microsyringe pump (World Precision Instruments). The infusions were performed for total four tracks of caudate head and anterior putamen (3 and 5 mm anterior to AC) in the left hemisphere of CR, and total 12 tracks of caudate head and anterior putamen (2, 4, and 6 mm anterior to AC) in the left and right hemispheres of DO (Table 1).

We started the recording of dLight signal in the caudate head and the anterior putamen 6 wk after the infusion. For recording the signal, we used a borosilicate mono fiber-optic cannula (200-μm core diameter, 0.66 NA, 130-mm length, RM2 receptacle, A60 taper tip; Doric). A low-autofluorescence mono fiber-optic patch cable (200-μm core diameter, 0.57 NA; Doric) was connected to the cannula with a connector (CM2). We used a 465-nm blue excitation light-emitting diode (LED) to excite dLight at 330 Hz frequency, and a 405-nm violet LED for isosbestic control at 210 Hz (2-channel LED Drivers; Doric). All two excitation signals were passed through a four-port filter cube (iFMC4; Doric), and emission signals were amplified using a fluorescence detector amplifier digitized at 15 kHz (FDA; Doric). The signals were sampled using a real-time signal processor (RZ5D; Tucker Davis Technologies) running the Synapse software suite. We progressed the cannula toward a target region while monitoring the change in the isosbestic control signal. After reaching the target region, we confirmed that the isosbestic control signal stayed regular and motion artifacts were negligible during the recording sessions (Figs. 2*A* and 3*A*).

Analysis of the dLight signals was performed using MATLAB (MathWorks). Neither downsampling nor smoothing methods were applied to the analysis. We calculated baseline *z*-score for normalizing the signals from dLight channel below.

$$Z_{i}=\left(x_{i}-\mu \right)/\sigma ,$$

where,

*μ* = mean of values from baseline period (1 s before CS onset)

*σ* = SD of values from baseline period

Peri-event histograms were then created by averaging changes in fluorescence across repeated trials during windows encompassing behavioral events of interest. Dunnett’s test was conducted for the normalized dLight signal during the subsequent delay period of CS event (0.1 to 0.6 s from CS offset). Tukey’s test was conducted for the normalized dLight signal during the post-US delivery period (0.5 to 1.0 s from US onset) in the probabilistic reward task.

To compare the dLight signal during the delay period of CS between two regions (anterior putamen and caudate head), each dLight response to CS was tested with the following linear regression model:

$$F_{\mathrm{CS}}=\beta_{0}+\beta_{1}\;RewardProbability,$$

with *F_CS_* as trial-by-trial dLight response to CS, *RewardProbability* as CS reward probability (P = 0, P = 0.5, and P = 1), *β*_0_ as constant, and *β*_1_ as corresponding regression coefficient. The regression coefficient *β*_1_ was estimated for each recording session. The Wilcoxon rank-sum test was performed to test for significant difference between the coefficients of the two regions.

To compare the dLight signals during the post-US delivery period between the two regions, each dLight response to US was tested with the following linear regression model:

$$F_{\mathrm{US}}=\beta_{0}+\beta_{1}\;PredictionError,$$

with *F_US_* as trial-by-trial dLight response to US, *PredictionError* as US prediction error (−1 was assigned to no-reward following P = 0.5 reward CS, 0 was assigned to both no-reward following P = 0 reward CS and reward following P = 1 reward CS, and +1 was assigned to reward following P = 0.5 reward CS), *β*_0_ as constant, and *β*_1_ as corresponding regression coefficient. The regression coefficient *β*_1_ was estimated for each recording session. The Wilcoxon rank-sum test was performed to test for significant difference between the coefficients of the two regions.

The two-sample *t* test was performed to test for significant difference between the normalized dLight responses of the two regions to the unpredicted reward delivery (0.7 to 1.2 s from unpredicted reward). All statistical tests were two-tailed.

### Histology.

After completing fluorescence and electrophysiological recordings (2 y and 6 mo after the vector infusion), one subject (monkey CR) was deeply anesthetized with an overdose of secobarbital sodium (25 mg/kg, i.v.) and perfused with 0.1 M phosphate-buffered saline (PBS; pH 7.4), followed by 10% formalin in 0.1 M phosphate buffer (PB) (73, 74). The brain was removed, post-fixed overnight at 4 °C, and saturated in 30% sucrose in 0.1 M PBS at 4 °C. Coronal sections were cut at 50-μm thickness using a freezing microtome (REM-710; Yamato Kohki Industrial). Every 10th section was mounted on a gelatin-coated glass slide and stained with 1% Cresyl violet (Nissl staining). Sections containing caudate head and anterior putamen were processed for immunofluorescence staining. These sections were washed once with 0.1 M PBS, then soaked in 1% skim milk for 1 h. They were incubated for 2 d at 4 °C with a rabbit monoclonal anti-GFP antibody (1:500 dilution, Invitrogen #G10362) and a mouse monoclonal anti-TH antibody (1:500 dilution, Millipore #MAB318) in 0.1 M PBS containing 1% normal donkey serum and 0.1% Triton X-100. After being washed three times in 0.1 M PBS, the sections were incubated at room temperature for 2 h with Alexa Fluor 488–conjugated donkey anti-rabbit IgG antibody (1:400 dilution; Invitrogen #A21206) and Alexa Fluor 555–conjugated donkey anti-mouse IgG antibody (1:400 dilution, Invitrogen #A31570) in freshly prepared medium. After three additional washes with 0.1 M PBS, the sections were mounted onto gelatin-coated glass slides, air dried, and covered with coverslips. Fluorescent images were captured using a fluorescence microscope (BZ-X800; Keyence).

### Neuronal Tracing.

For retrograde tracing, dual-color retrograde vectors (rAAV2-retro-hSyn-mScarlet and rAAV2-retro-hSyn-AcGFP) (75) were infused into the caudate head and the anterior putamen of two subjects (monkeys CN and DK) (Table 1). Each vector (4.0 × 10^13^ genome copies/mL) was unilaterally infused into either the caudate head or the anterior putamen in the left hemisphere using an MRI-guided navigation system (Brainsight Primate, Rogue Research) (73, 74). The infusion was performed at a rate of 0.4 μL/min (2.5 μL per site, two sites per track) using a 10-μL Hamilton syringe. For each subject, one track was targeted in the caudate head (5 mm anterior to AC) and one track in the anterior putamen (5 mm anterior to AC) (Table 1). Following the infusions, the scalp incision was sutured.

### Tissue Processing.

After a 4-wk survival period, each subject was deeply anesthetized with an overdose of secobarbital sodium (25 mg/kg, i.v.) and transcardially perfused with 0.1 M PBS, followed by 10% formalin in 0.1 M PB. The brain was then removed, post-fixed overnight at 4 °C, and saturated with 30% sucrose in 0.1 M PBS at 4 °C. Coronal sections were cut serially at a thickness of 50 μm using a freezing microtome (REM-710; Yamato Kohki Industrial). Every 10th section was mounted onto a gelatin-coated glass slide and stained with 1% Cresyl violet (Nissl staining).

### TH Immunofluorescence.

Sections containing the substantia nigra were washed once in 0.1 M PBS, immersed in 1% skim milk for 1 h, and incubated for 2 d at 4 °C with a mixture of primary antibodies: rabbit monoclonal anti-GFP (1:1,000 dilution; Invitrogen #G10362), rat monoclonal anti-RFP (1:500 dilution; Chromotek #5F8), mouse monoclonal anti-TH (1:500 dilution; Millipore #MAB318), and guinea pig polyclonal anti-NeuN (1:500 dilution; Millipore #ABN90). The antibodies were diluted in 0.1 M PBS containing 1% normal donkey serum and 0.1% Triton X-100. After three washes in 0.1 M PBS, the sections were incubated for 2 h at room temperature with secondary antibodies: Alexa Fluor 488–conjugated donkey anti-rabbit IgG (1:400 dilution; Invitrogen #A21206), Cy3-conjugated donkey anti-rat IgG (1:200 dilution; Jackson laboratories #712-165-153), Alexa Fluor 647–conjugated donkey anti-mouse IgG (1:200 dilution; Invitrogen #A31571), and DyLight 405–conjugated donkey anti-guinea pig IgG (1:200 dilution; Jackson laboratories #706-475-148) in the same fresh incubation medium. Following three additional washes in 0.1 M PBS, the sections were mounted on gelatin-coated glass slides and coverslipped. Fluorescent labeled cells in each coronal section were plotted using Neurolucida software (MicroBrightField; Bioscience). Fluorescent images were captured using a fluorescence microscope (BZ-X800; Keyence).

### Test of dLight Signal in Rats.

Two wild-type Long-Evans rats (male, 9 wk old, 297 to 315 g) were used for testing dLight signals. These animals were kept in their home cage under an inverted light schedule (lights off at 9 am, lights on at 9 pm). A rat was conditioned in a Pavlovian procedure with its head fixed to a stereotaxic frame. Behavioral control and data acquisition were conducted by a real-time signal processor (RZ5D; Tucker Davis Technologies) running the Synapse software suite. During the first block of trials, a 10-kHz tone lasting for 0.3 s was followed by the delivery of water reward (5 μL × 3 times in 0.6 s) with a delay of 0.3 s. The trial was transitioned to the second block without any external cue, and the tone was not followed by any water reward.

#### Fiber photometry.

The rats were handled by the experimenter (5 min, twice) in advance. For head-plate (CFR-1, Narishige) implantation, the animals were anesthetized with isoflurane through inhalation anesthesia apparatus (5% for induction, 2.0 to 2.5% for maintenance) and were placed on a stereotaxic frame (SR-10R-HT, Narishige). The plate was surgically attached to their skulls with tiny anchor screws (M1, 2-mm long, stainless steel) and dental acrylic resin (Unifast II, GC Corporation; Estecem II, Tokuyama Dental Corporation). Lidocaine (Xylocaine jelly 2%) was administered around the surgical incisions. During anesthesia, body temperature was maintained at 37 °C using an animal warmer (BWT-100, Bio Research Center). Analgesic (Meloxicam, 1 mg/kg s.c.) and antibiotic (Chlomy-P ointment) were applied postoperatively as required. After 1 wk of recovery period, the rats had ad libitum access to water during weekends, but they obtained water during the task on weekdays. When necessary, agar was given to them in their home cage to maintain their body weights.

A total of 1 μL of AAV2.1-CaMKIIα-dLight1.1 (1.0 × 10^13^ genome copies/mL) was infused into each site in the striatal regions (the DMS and ventral striatum; AP: 0.0 mm, ML 3.4 mm, DV: 4.5 and 6.9 mm) based on a rat brain atlas (76) at a speed of 0.1 μL/min using a glass microinjection capillary connected to a microinfusion syringe pump (Legato100; KD scientific), and the capillary was left in the place for 5 min after the infusion. We started the recording of dLight signal 3 wk after the infusion. For recording the signal, we used a borosilicate mono fiber-optic cannula (200-μm core diameter, 0.66 NA; Doric). The data acquisition system and procedures were the same as in monkeys.

#### Histology.

The rats were deeply anesthetized with an overdose of sodium pentobarbital (100 mg/kg, i.v.) and perfused transcardially with 0.1 M PBS, followed by 10% formalin in 0.1 M PB. The brains were post-fixed overnight at 4 °C and saturated with 30% sucrose in 0.1 M PBS at 4 °C. Frozen brains were sectioned at a thickness of 40 μm using a microtome (REM-710; Yamato Kohki Industrial).

The sections were washed once in 0.1 M PBS, immersed in 1% skim milk for 1 h, and incubated for 2 d at 4 °C with the following primary antibodies: chicken polyclonal anti-GFP (1:500 dilution, Abcam #ab13970), mouse monoclonal anti-TH (1:500 dilution, Millipore #MAB318), and rabbit monoclonal anti-NeuN (1:500 dilution, Millipore #ab177487). These antibodies were diluted in 0.1 M PBS containing 1% normal donkey serum and 0.1% Triton X-100. After three washes in 0.1 M PBS, the sections were incubated for 2 h at room temperature with secondary antibodies: Alexa Fluor 488–conjugated goat anti-chicken IgY (1:200 dilution, Millipore #A11039), Alexa Fluor 647–conjugated donkey anti-mouse IgG (1:200 dilution, Jackson laboratories #A31571), and Alexa Fluor 350–conjugated donkey anti-rabbit IgG (1:200 dilution, Invitrogen #A10039) in the same fresh medium. After three additional washes in 0.1 M PBS, the sections were mounted onto gelatin-coated glass slides, air dried, and coverslipped. Fluorescent images were captured using a fluorescence microscope (BZ-X800; Keyence).

## Supplementary Material

Appendix 01 (PDF)

Dataset S1 (XLSX)

## Data Availability

All study data are included in the article and/or *SI Appendix*.
