# Supplementary material for: Fluorescence detection of dopamine signaling to the primate striatum in relation to stimulus–reward associations
Source: Proc Natl Acad Sci U S A. 2025 Mar 13;122(11):e2426861122. doi: 10.1073/pnas.2426861122 (PMC11929443; doi:10.1073/pnas.2426861122)
Supplement: Supplementary file 1 — Appendix 01 (PDF) [file pnas.2426861122.sapp.pdf]

PNAS

Supporting Information for

Fluorescence detection of dopamine signaling to the primate striatum in relation to stimulus-reward associations

Gaoge Yan, Hidetoshi Amita*, Satoshi Nonomura, Ken-ichi Inoue, Wolfram Schultz, Masahiko Takada*

*Corresponding authors: Hidetoshi Amita and Masahiko Takada

Email: amita.hidetoshi.7x@kyoto-u.ac.jp (H.A.)

This PDF file includes:

Figures S1 to S2

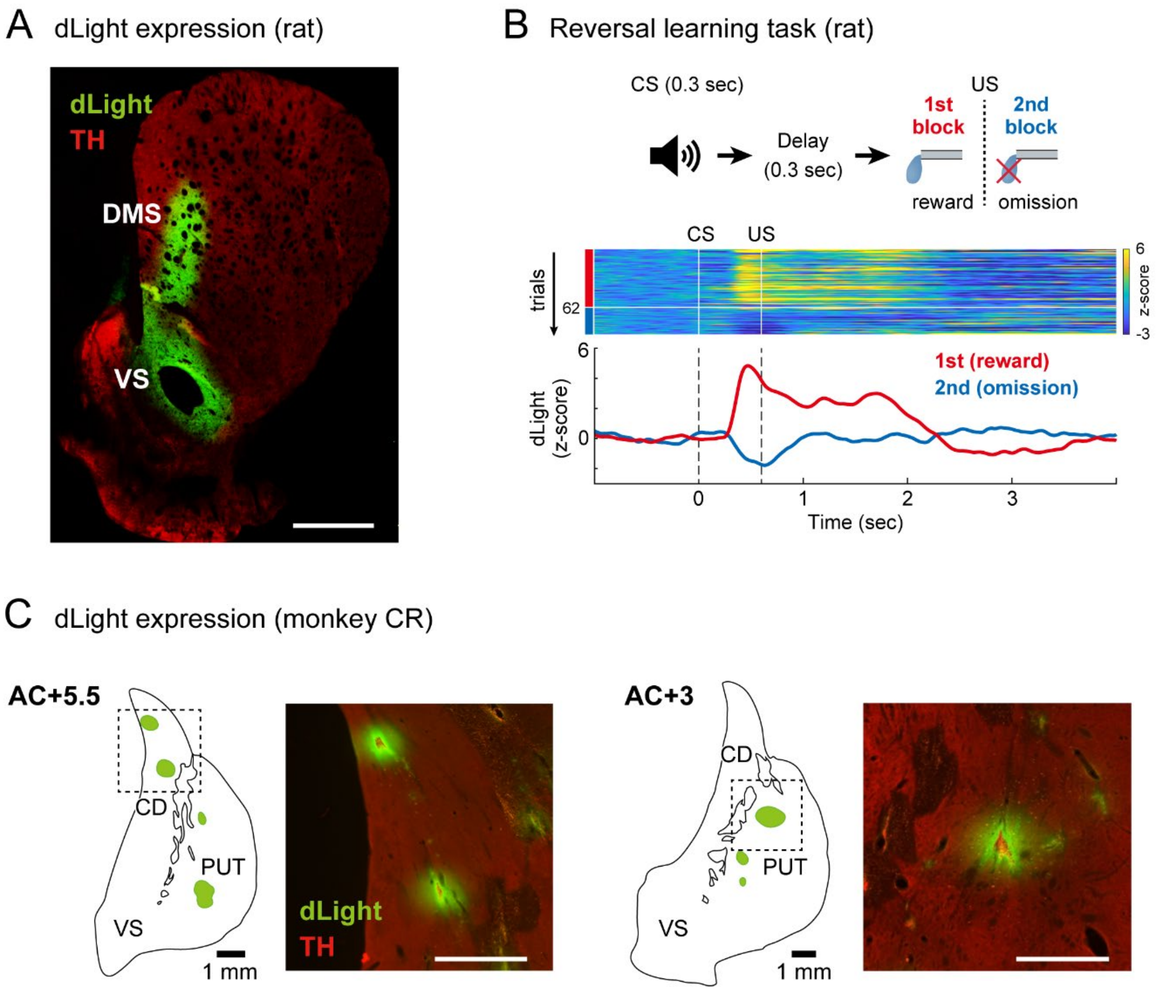

Fig. S1. Expression of dLight1.1 in the striatal regions of a rat and a monkey

(*A*) Sites of dLight1.1 expression in the rat striatum. dLight1.1 (green) was expressed in the dorsomedial striatum (DMS) and the ventral striatum (VS). The striatum was immunostained for TH (red). Scale bar, 1 mm. (*B*) Average normalized dLight signal in the rat striatum during the reversal learning task (90 trials in total). CS (auditory stimulus) was followed by reward in the first block (red). After 62 trials, the reward was omitted in the second block (blue). (*C*) Sites of dLight1.1 expression in the caudate head (CD) and anterior putamen (PUT) of monkey CR. Green areas in drawings (*Left*) indicate dLight1.1 expression in coronal sections 5.5 and 3 mm anterior to the anterior commissure (AC+5.5 and AC+3, respectively). Captured images (*Right*) are taken from dotted squares in the drawings. The striatum was immunostained for TH (red). Scale bar, 1 mm.

A Injection sites (monkey DK)

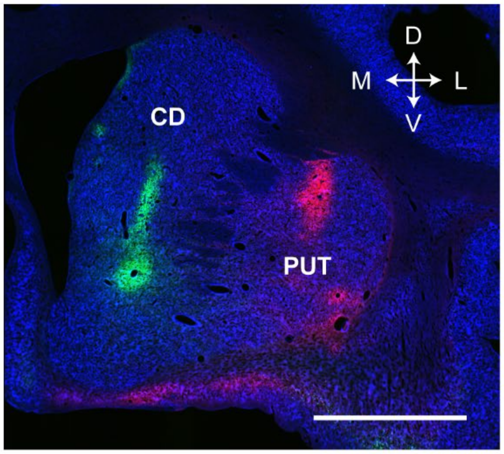

B TH-positive cells labeled with GFP/RFP

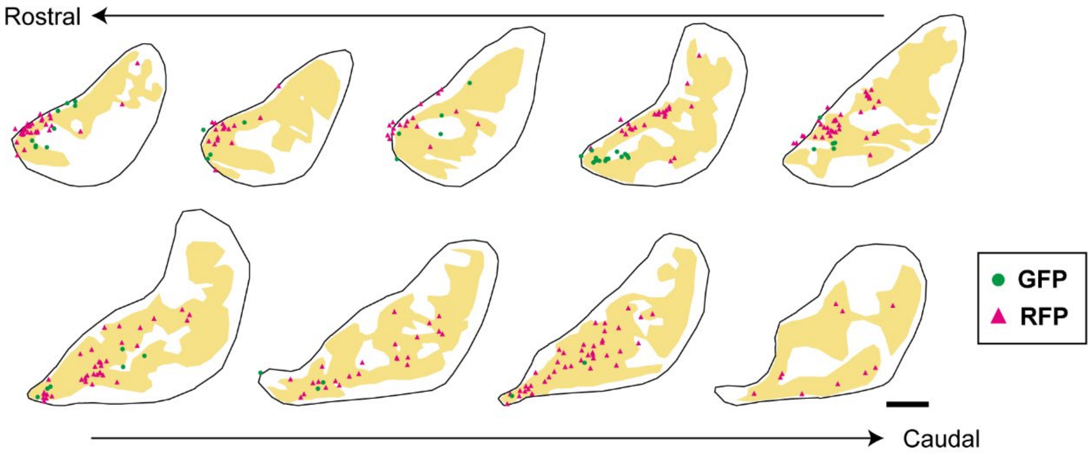

Fig. S2. Distribution of TH-positive cells projecting to the caudate head vs. the anterior putamen.

(*A*) Injection sites in a representative coronal section (monkey DK). rAAV2-retro-GFP (green) and rAAV2-retro-RFP (red) vectors were injected into CD and PUT, respectively. A combination of the vector and its injection site was reversed from that in monkey CN (see Fig. 5). Scale bar, 5 mm. (*B*) Distribution of TH-positive cells in substantia nigra pars compacta (SNc) that were labeled with GFP/RFP. Green and red symbols denote cells labeled with GFP and RFP, respectively. The yellow shaded area demarcates the SNc. Coronal sections 500-μm apart are arranged rostrocaudally. Scale bar, 1 mm.
